# Thoracic endovascular aortic repair for acute aortic dissection complicated by mesenteric malperfusion: an evaluation by computational fluid dynamics

**DOI:** 10.1093/icvts/ivae047

**Published:** 2024-03-18

**Authors:** Naoyuki Kimura, Shuta Imada, Daijiro Hori, Masanori Nakamura

**Affiliations:** Department of Cardiovascular Surgery, Saitama Medical Center, Jichi Medical University, Saitama, Japan; Department of Electrical and Mechanical Engineering, Nagoya Institute of Technology, Nagoya, Japan; Department of Cardiovascular Surgery, Saitama Medical Center, Jichi Medical University, Saitama, Japan; Department of Electrical and Mechanical Engineering, Nagoya Institute of Technology, Nagoya, Japan

**Keywords:** Mesenteric malperfusion, Thoracic endovascular aortic repair, Computational fluid dynamics

## Abstract

Computational fluid dynamics was performed to simulate haemodynamics of type B aortic dissection complicated by mesenteric malperfusion caused by dynamic obstruction in a 70-year-old man. Streamline analysis showed disappearance of antegrade flow in the false lumen of the descending aorta and attenuation of intermittent flap-induced disruption of visceral vessel perfusion after entry coverage. Quantitative analysis showed endovascular repair increased perfusion volume of the coeliac artery and superior mesenteric artery by 55.6% and 77.4%, respectively. Entry closure with thoracic endovascular prosthesis improved mesenteric malperfusion by attenuating the intermittent flap-induced perfusion disruption.

## INTRODUCTION

Mesenteric malperfusion is caused by obstruction, whether dynamic, static or both, of the superior mesenteric artery. In cases of dynamic obstruction, thoracic endovascular aortic repair (TEVAR) can restore blood flow in the true lumen (TL) by covering the entry tear and any other communication between the TL and the false lumen (FL).

Computational fluid dynamics (CFD) has been used to investigate efficacy of TEVAR in patients with acute aortic dissection (AAD) [1]. However, CFD-based studies of haemodynamic change after TEVAR for organ malperfusion have been limited [2]. We performed a CFD simulation to investigate haemodynamic change in an AAD patient complicated by mesenteric malperfusion, aiming to clarify therapeutic mechanism of TEVAR.

## CASE REPORT

A 70-year-old man was admitted for type B AAD 6 h after the onset of abdominal pain. Computed tomography revealed an entry tear and narrowed TL in the descending aorta, but the dissection did not extend to the visceral vessels (Supplementary Material, Fig. S1). Type B AAD complicated by mesenteric malperfusion due to dynamic obstruction was suspected. The patient was initially treated conservatively. However, paralytic ileus persisted. TEVAR was performed on hospital day 12. Operative information is shown in the Supplementary Material. Postoperative computed tomography showed an expanded TL and thrombosed FL (Supplementary Material, Fig. S1). The ileus improved postoperatively, and the patient resumed oral ingestion. Although spinal cord injury had occurred, paralysis improved to the level of wheelchair use.

**Figure 1:**
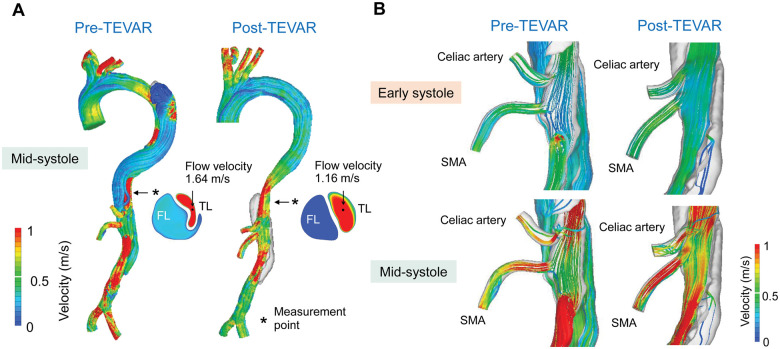
Streamline analysis. (**A**) Aorta at the mid-systole. (**B**) Visceral vessels at the early- and mid-systole. FL: false lumen; TEVAR: thoracic endovascular aortic repair; TL: true lumen.

CFD information is shown in Supplementary Material, Fig. S2. Aortic flow patterns at mid-systole are shown in Fig. 1A and Video 1. After TEVAR, the antegrade blood flow in the FL disappeared. Accompanied by TL expansion, flow velocity in the TL decreased from 1.64 to 1.16 m/s at the level of diaphragm (Fig. 1A). Streamline patterns of the visceral vessels are shown in Fig. 1B and Video 2. Before TEVAR, perfusion of the visceral vessels was repeatedly disrupted, caused by intermittent covering of the vessel’s orifice by the intimal flap. After TEVAR, the disruption in visceral perfusion was attenuated, and blood flow to the visceral vessels increased during systole (Fig. 1B).

**Figure 2:**
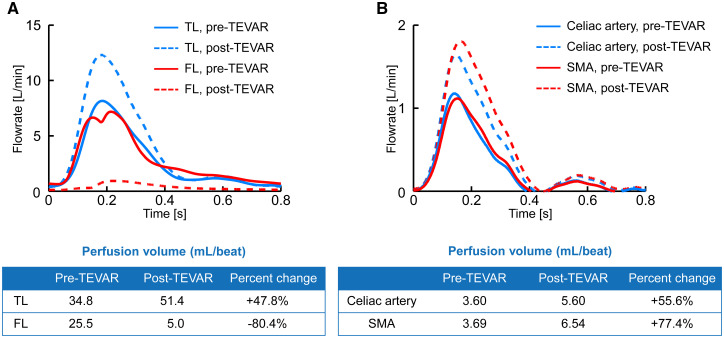
Line graph showing flow rate and perfusion volume before and after TEVAR. (**A**) TL and FL of the descending aorta. (**B**) Visceral vessels. SMA (superior mesenteric artery); TEVAR: thoracic endovascular aortic repair; Celiac artery.

Flow rate and perfusion volume of the descending aorta are shown in Fig. 2A. After TEVAR, flow rate in the TL at the level of diaphragm increased, but the flow rate in the FL was remarkably decreased. Perfusion volume in the TL increased from 34.8 to 51.4 ml/beat. Alternatively, perfusion volume in the FL decreased from 25.5 to 5.0 ml/beat. Changes in flow rate and perfusion volume of the visceral vessels are shown in Fig. 2B. After TEVAR, perfusion volume in the coeliac artery increased from 3.6 to 5.6 ml/beat (a 55.6% increase), and perfusion volume in the superior mesenteric artery increased from 3.69 to 6.54 ml/beat (a 77.4% increase).

## DISCUSSION

Dynamic obstruction accounts for 80% of cases of malperfusion associated with AAD [3]. There are no reported studies in which visceral blood flow was measured after TEVAR for AAD with mesenteric malperfusion. Our data showed FL depressurization achieved by TEVAR resulted in TL expansion and increased blood flow to the visceral vessels. Also, TEVAR increased perfusion volume of the visceral vessels by nearly 50%. Future study including a large number of patients should be conducted to validate our findings.

## Supplementary Material

ivae047_Supplementary_Data

## Data Availability

All relevant data are within the manuscript and its Supporting Information files.
