# Corrigendum to: Thoracic endovascular aortic repair for acute aortic dissection complicated by mesenteric malperfusion: an evaluation by computational fluid dynamics

**DOI:** 10.1093/icvts/ivaf134

**Published:** 2025-06-16

This is a corrigendum to: Naoyuki Kimura, Shuta Imada, Daijiro Hori, Masanori Nakamura, Thoracic endovascular aortic repair for acute aortic dissection complicated by mesenteric malperfusion: an evaluation by computational fluid dynamics, *Interdisciplinary CardioVascular and Thoracic Surgery*, Volume 38, Issue 3, March 2024, ivae047, https://doi.org/10.1093/icvts/ivae047

The authors identified an error in the reported stroke volume used in their CFD simulations. The originally stated stroke volume of 66.4 mL/beat was incorrect; the correct value is 83.0 mL/beat. This correction affects the description in the **Supplementary Methods** section, the **Case Report** section of the main text, and numerical values shown in Figure 2. Additionally, there were labeling errors in the figure.

The following emendations are needed:

In the **Supplementary Methods** section revised text should read: “A physiological flow rate with a heart rate of 75 bpm and stroke volume of 83.0 mL/beat at the aortic root was assigned as the inlet boundary condition.” (The emended file is attached to this notice.)

In **Case Report** section of the main text, revised text should read: “Perfusion volume in the TL increased from 34.8 mL/beat to 51.4 mL/beat. Alternatively, perfusion volume in the FL decreased from 25.5 mL/beat to 5.0 mL/beat. Changes in flow rate and perfusion volume of the visceral vessels are shown in Fig. 2B. After TEVAR, perfusion volume in the coeliac artery increased from 3.6 mL/beat to 5.6 mL/beat (a 55.6% increase), and perfusion volume in the super mesenteric artery increased from 3.69 mL/beat to 6.54 mL/beat (a 77.4% increase).”

Figure 2 should read:
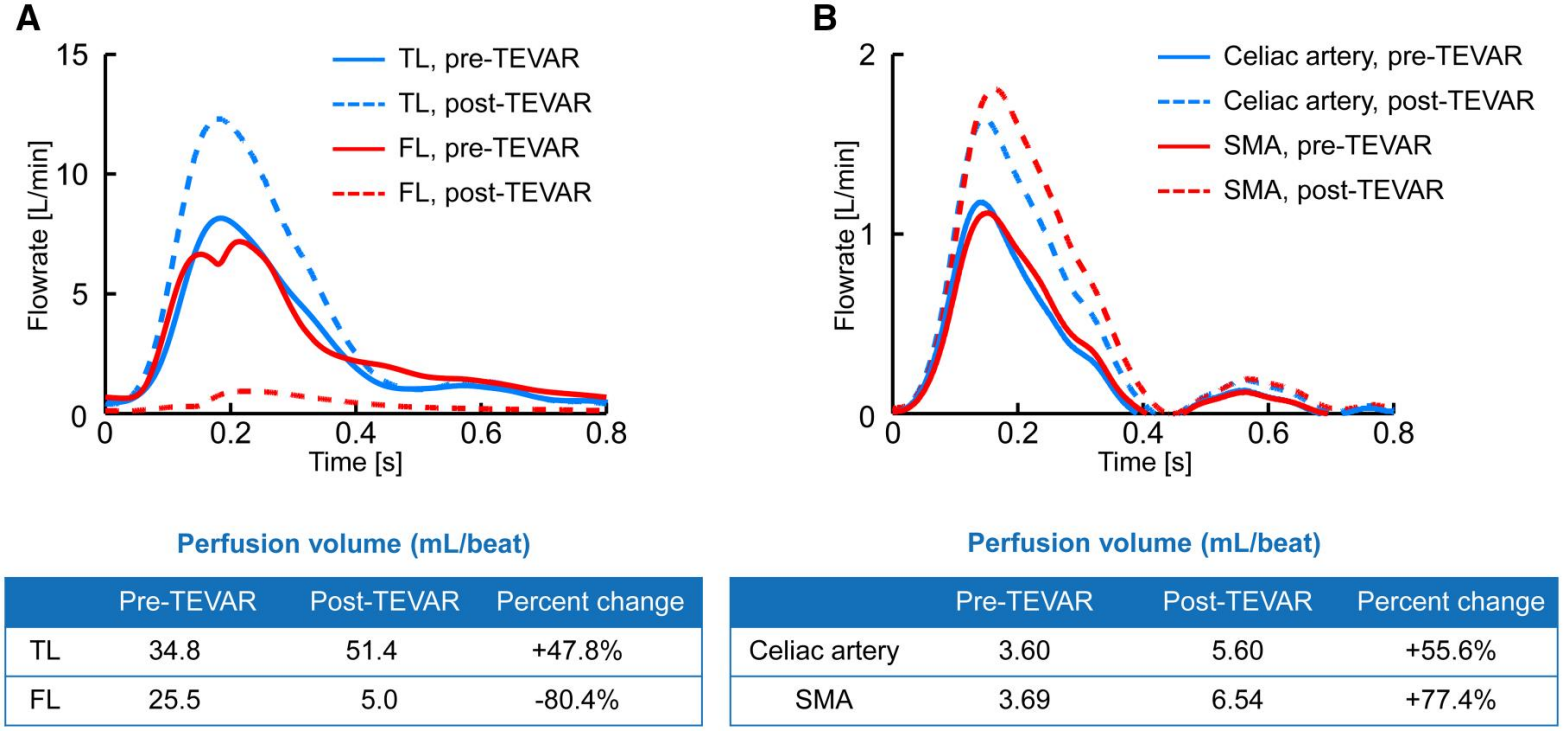


which in Panel B reflects the updated numerical data. There are typographical errors in the figure labels for 3B also. “TL” should read: “Celiac artery”; “FL” should read “SMA”. The affected part of legend for **Figure 2** should read:

**“**[…](**B**) Visceral vessels. SMA (superior mesenteric artery); TEVAR: thoracic endovascular aortic repair; Celiac artery.”

Please note these corrections do not affect the conclusions of the paper. The emendations are outlined only in this notice to preserve the version of record.

