# Supplementary material for: Thoracic endovascular aortic repair for acute aortic dissection complicated by mesenteric malperfusion: an evaluation by computational fluid dynamics
Source: Interdiscip Cardiovasc Thorac Surg. 2024 Mar 18;38(3):ivae047. doi: 10.1093/icvts/ivae047 (PMC11095047; doi:10.1093/icvts/ivae047)
Supplement: ivae047_Supplementary_Data [file ivae047_Supplementary_Data.docx]

**Supplementary Data**

**METHODS**

**Operative procedure**

TEVAR was performed to close the primary entry tear, located at the mid-descending thoracic aorta. Under general anesthesia, a stiff guidewire was inserted into the ascending aorta via the right femoral artery after confirmation of the TL by intravascular ultrasound (IVUS). Size of the TL was confirmed by IVUS, with the proximal and distal diameters shown to be 30 mm and 26 mm, respectively. A TX-D Extension (diameter 26 mm, length 80 mm; Cook Medical, Bloomington, IN, USA) was deployed at the straight portion of the descending thoracic aorta, after which a tapered Relay Thoracic Stent-Graft (diameter 32-28 mm, length 164 mm; Terumo Aortic, Sunrise, FL, USA) was placed just distal to the origin of subclavian artery, covering the primary entry tear. Because of type III endoleak, a 34-mm Relay (length 150 mm) was added to secure the junction between the two stent grafts.

**Computer simulation of blood flow**

CT angiograms had been obtained preoperatively and postoperatively with a SOMATOM Definition Flash scanner (Siemens Healthcare, Munich, Germany). The scanning had been performed at a slice thickness of 1 mm. Analysis was performed with Synapse Vincent (Fujifilm Medical, Tokyo, Japan). The CT scan covered the entire aorta including the 3 main branches arising from the aortic arch and the origin of common iliac arteries.

The luminal geometries of the aorta, celiac artery, and SMA were extracted from the preoperative and postoperative CT angiograms, which had a spatial resolution of 0.877 mm x 0.877 mm x 0.5 mm and 0.782 mm x 0.782 mm x 0.5 mm, respectively. The geometries were reconstructed with 3D Slicer 4.10.2 open-source imaging software. The created geometries were confirmed by well-experienced cardiac surgeons. A 2-layer surface prism mesh was generated, and a polyhedral mesh was generated for the internal region. The total number of mesh elements was approximately 860,000.

Pulsatile blood flow in the aorta was simulated with a commercially available CFD program (scFLOW v2021, MSC Software Japan, Tokyo, Japan). Multiscale blood flow simulation was achieved by coupling the Navier-Stokes equations in a 3D domain to peripheral blood flow models in a 0D domain (**Fig. S2**). Peripheral blood flow modeling was based on the hydraulic-electric analogue, in which vascular resistance, pressure difference, and mass flow are represented by electrical resistance, potential difference, and electrical current, respectively. Vascular resistance *R* was determined on the basis of a structured tree model [5-7]. Pressure at the downstream end of the 0D model was taken as capillary pressure, *P_t_* = 30 mmHg [6]. A physiological flow rate with a heart rate of 75 bpm and stroke volume of 66.4 mL/beat at the aortic root was assigned as the inlet boundary condition. The wall was assumed to be rigid, and a no-slip boundary condition was applied. Blood was assumed to be an incompressible Newtonian fluid, with non-Newtonian behavior being negligible in large arteries. Density and dynamic viscosity were set to 1.05 x 10^3^ kg/m^3^ and 4.0 x 10^-3^ Pa/s, respectively.

Hemodynamic changes in flow pattern effected by TEVAR were investigated by streamline analysis. Flow rate [L/min] and perfusion volume/cardiac cycle were calculated at (1) both the TL and FL of the descending thoracic aorta at the diaphragm, (2) celiac artery, and (3) SMA (**Fig. S2**).

**
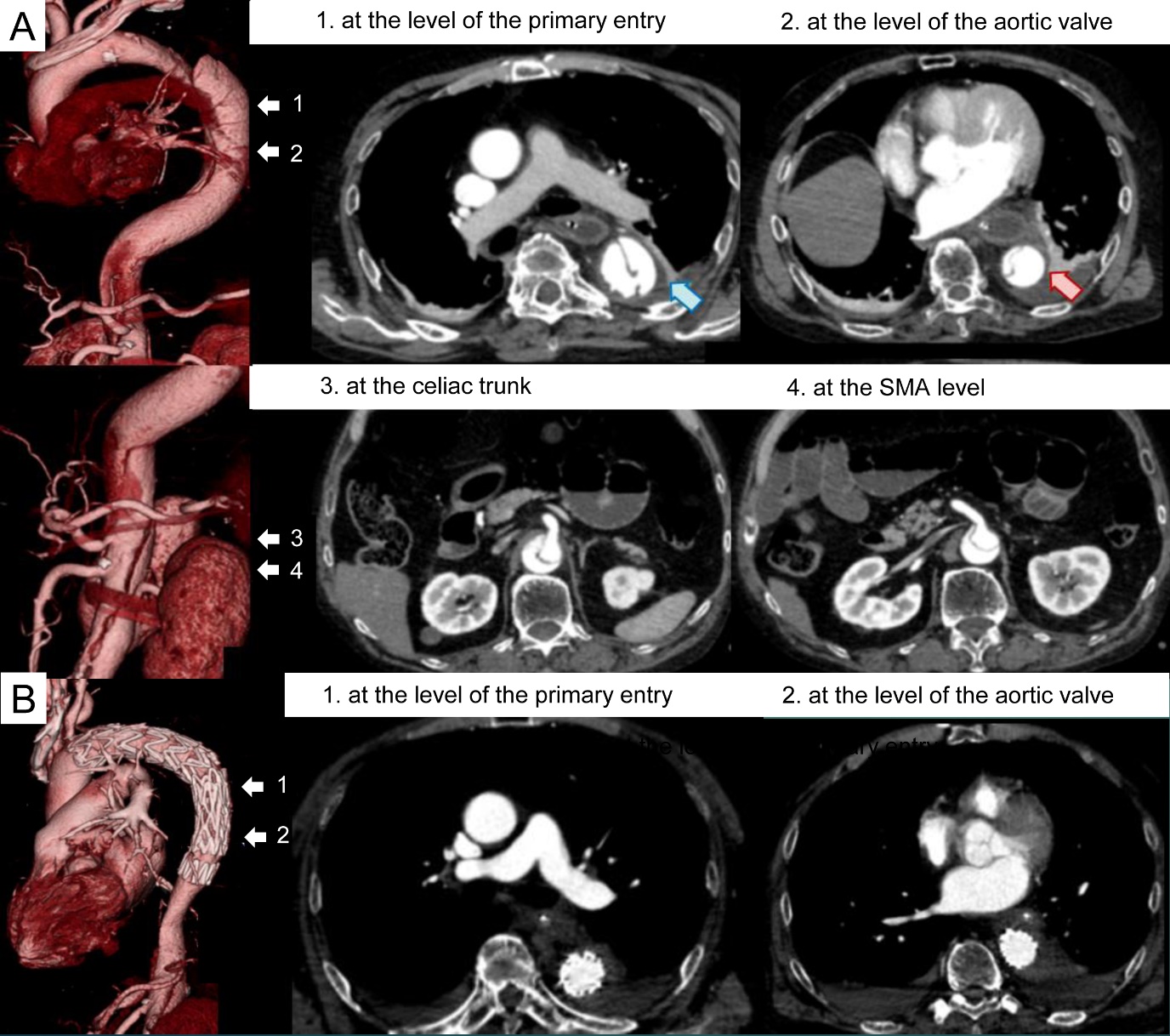
Fig. S1.**

**Fig. S1:** CT angiography images obtained during examination of a patient with acute type B aortic dissection complicated by mesenteric malperfusion. (A) Preoperative CT images. (B) Postoperative CT images. Blue arrow indicates the primary entry tear. Red arrow indicates the narrowed true lumen in the descending thoracic aorta. CT: computed tomography; SMA: superior mesenteric artery.

**Figure S2.**


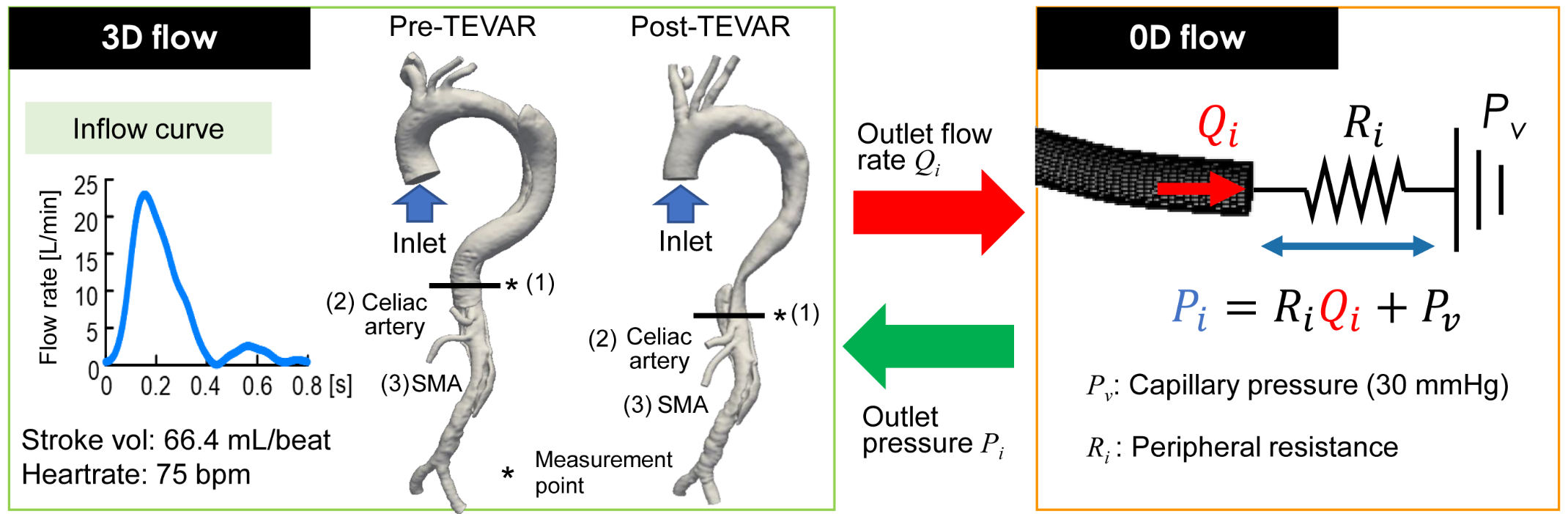


**Fig. S2:** Procedural steps for patient-specific CFD simulation of blood flow. CFD: computational fluid dynamics; SMA: superior mesenteric artery; TEVAR: thoracic endovascular aortic repair.
